# Intraepithelial CD8^+^ T-cell-count becomes a prognostic factor after a longer follow-up period in human colorectal carcinoma: possible association with suppression of micrometastasis

**DOI:** 10.1038/sj.bjc.6602201

**Published:** 2004-10-19

**Authors:** T Chiba, H Ohtani, T Mizoi, Y Naito, E Sato, H Nagura, A Ohuchi, K Ohuchi, K Shiiba, Y Kurokawa, S Satomi

**Affiliations:** 1 Division of Advanced Surgical Science and Technology, Tohoku University Graduate School of Medicine, Sendai, Japan; 2 Department of Pathology, Tohoku University Graduate School of Medicine, Sendai, Japan; 3 Department of Pathology, Mito Medical Center, Ibaraki, Japan; 4 Division of Biological Regulation and Oncology, Department of Surgery, Tohoku University Graduate School of Medicine, Sendai, Japan; 5 Department of Surgery, Tohoku Rosai Hospital, Sendai, Japan; 6 Department of Surgery, Miyagi Cancer Center, Natori, Japan

**Keywords:** colorectal cancer, intratumoral CD8+ T cells, multivariate analyses, tumour immunity

## Abstract

T-cell infiltration into human cancer tissues can be a manifestation of host immune responses to cancer cells. The present study was undertaken to explore the clinicopathological significance of intraepithelial CD8^+^ T cells using 371 consecutively sampled human colorectal carcinomas. By univariate analysis, we noted that the survival curves by intraepithelial CD8^+^ T cells became separated only after 1 to 2 years postoperation. Multivariate analyses revealed that the beneficial effect of this factor becomes significant only after a longer (more than 2 year), but not after a shorter (less than 2 year) follow-up period. Furthermore, the number of intraepithelial CD8^+^ T cells was significantly higher in patients alive for more than 5 years than in patients who either died of cancer after a curative operation or patients who underwent a noncurative operation. Patients' cancer-specific death long after a curative operation is thought to be caused by the growth of micrometastases in other organs or near the primary sites. The effects of intraepithelial CD8^+^ T cells, therefore, may be mediated by suppression of micrometastasis, rather than suppression of growth in the primary tumour. In conclusion, our data support a hypothesis on the presence of systemic immunosurveillance against micrometastasis of cancer cells.

After considerable controversy, recent studies have supported the hypothesis that the presence of immunosurveillance has an effect on tumour development (Smyth *et al*, 2001; Dunn *et al*, 2002). Paraneoplastic syndromes associated with degeneration of nervous tissues are now regarded as a manifestation of effective antitumour immune responses against neuronal antigens expressed by tumour cells (Albert and Darnell, 2004). Tumour-associated antigens recognised by T cells have been reported, and therapeutic trials utilizing tumour vaccines are now ongoing (Henderson and Finn, 1996; Melief *et al*, 2000; Rosenberg 2001). On the other hand, tumour cells protect themselves from the host's surveillance by utilizing various immune evasion mechanisms (Khong and Restifo, 2002). Thus, immunocompetent and cancer cells interact mutually forming a complex relationship.

As for colorectal cancer, Naito *et al* (1998) have pointed out in a study of 131 patients that the number of intraepithelial CD8^+^ T cells (designated as *CD8*^*+*^*T cells within cancer cell nests* in this paper) has a more significant impact on patients' survival than that of CD8^+^ T cells in other locations within the tumour. Recently, Menon *et al* (2004) pointed out that CD8^+^ and CD57^+^ cells along the invasive margin have a significant impact on better disease-free survival in patients with colorectal cancer in a study of 93 patients. CD8^+^ T cells distributed among cancer cells or along the invasive margin are positively associated with apoptosis of cancer cells in hepatocellular (Fukuzawa *et al*, 2001) and oesophageal carcinomas (Schumacher *et al*, 2001). Various immune cell responses in ovarian carcinomas at stages III and IV are also associated with better prognosis (Zhang *et al*, 2003). In renal cell carcinoma, however, Nakano *et al* (2001) clearly showed that intratumoral CD8^+^ T cells (defined as *CD8*^*+*^*T cells among cancer cells or those in contact with cancer cells*), as well as CD8^+^ or CD4^+^ lymphocytes in other cancer locations, were positively correlated with the biological malignancy of cancer, associating lymphocyte responses with a poor prognosis. Furthermore, 10–20% of human colorectal carcinoma is associated with microsattelite instability (MSI), and cases with MSI are characterised by more frequent infiltration by lymphocytes, increased rate of apoptotic cancer cells, and better survival than cases with microsatellite stability (MSS) (Jass *et al*, 1995; Dolcetti *et al*, 1999; Michael-Robinson *et al*, 2001; Ishikawa *et al*, 2003). These complexities led us to revisit our previous analyses in a larger-scale study. Here we show that the effects of intraepithelial CD8^+^ T cells become manifest after longer follow-up periods, allowing us to speculate that host immune responses represented by local CD8^+^ T-cell infiltration may actually contribute to the patients' prognosis by suppressing micrometastases.

## MATERIALS AND METHODS

### Patients (Table 1)

**Table 1:** Peritumoral TILs defined as mononuclear cell infiltrate along the invasive margin

**Age**		**Gender**		**Stage**		**Site**		**Invasion**^a^		**Peritumoral TILs**^a^		**Differentiation**	
Mean	63.2	Male	214	I	51	Right-sided colon	117	Expanding	211	Continuous	60	Poorly differentiated	22
s.d.	11.3	Female	157	II	137	Left-sided colon	113	Infiltrating	160	Others	311	Others	349
Median	63			III	136	Rectum	141						
				IV	47								

a Defined as Jass *et al* (1987).

The present study is a retrospective study using a total of 371 cases of colorectal carcinoma including all stages, which were consecutively sampled from Tohoku Rosai Hospital (Sendai), Tohoku University Hospital (Sendai), and Miyagi Cancer Center Hospital (Natori). All cases underwent resection of the primary tumour. Cases with macroscopic or microscopic residue of tumour cells at the surgical margins and those with preoperative chemo- or irradiation therapy were excluded. The average age was 63.2 years (s.d. 11.3), ranging from 28 to 88, male to female ratio 214 : 157. The average follow-up period of surviving patients was 2674 days (7.7 years) with an s.d. of 1403 days. All cases were histopathologically diagnosed according to the WHO classification (Hamilton and Aaltonen, 2000) and TNM staging (Sobin and Wittekind, 2002). The number of cases of TNM stages I, II, III, and IV, corresponding to Dukes A, B, C, and D, were 51, 137, 136, and 47, respectively. Lymph node metastases were checked by histopathological examination in all cases. Distant metastases were diagnosed by either CT scanning or histopathological examination. ‘Curative operation’ here means that the patient has no clinically detectable reside or metastasis at the time of surgical resection of the primary tumour. Cancer-specific death was recorded in 74 cases in this group.

### Histopathology and immunohistochemistry

Formalin-fixed, paraffin-embedded sections were used. For immunohistochemistry, we used the following primary antibodies: anti-CD8 (clone C8/144B, Dakocytomation Japan, Kyoto, Japan), anti-hMLH1, and anti-hMSH2. After deparaffinisation, sections were pretreated by autoclave heating for 5 min. Dextran-polymer method with EnVison plus (Dako) was used as the secondary antibody.

### Methods of cell counting

Intraepithelial CD8^+^ T cells were defined as CD8^+^ T cells located within cancer cell nests, not including those in the stroma, and their distribution was quantified as described previously (Nakano *et al*, 2001). In brief, we first observed the whole area of specimens to select three areas with the most abundant infiltration of intraepithelial CD8^+^ T cells. Areas near necrosis were excluded. Cell counting was performed using an ocular grid at × 200 magnification. All the counting was carried out by two independent observers, and the correlation coefficiency between the data obtained by the two observers was 0.96.

### Statistical analyses

The impact on the cancer-specific survival rate was tested by uni- and multivariate analyses with SPSS 11.0J. (SPSS Japan Inc., Tokyo, Japan). The observation periods were divided at 2 years in the multivariate analyses, because the number of patients who died of the primary disease became equal with this dichotomy.

### Double-labelling immunohistochemisty for CD8 and Ki-67

The method was described previously (Naito *et al*, 1998). The labelling index for Ki-67 in CD8+ T cells was obtained by the ratio of the number of double positive cells per the sum of number of single CD8+ T cells and that of double positive cells

## RESULTS

Figure 1A

**Figure 1:**
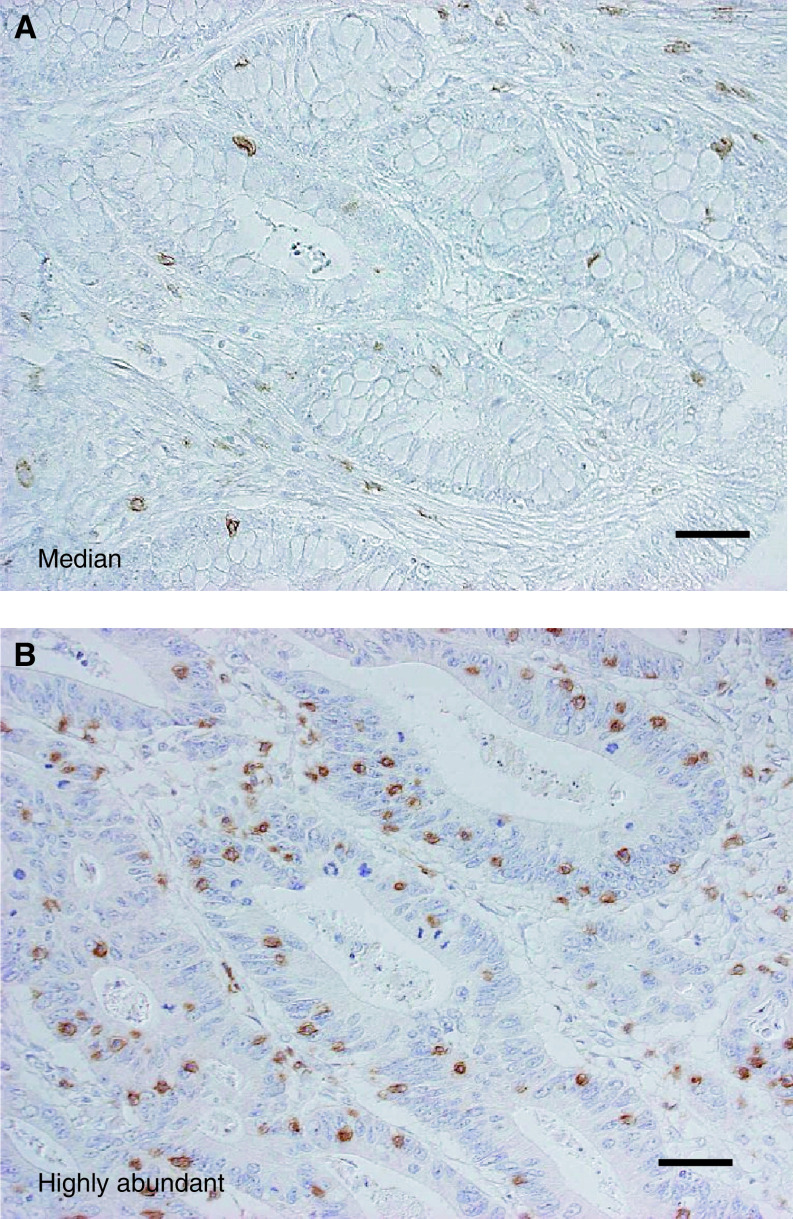
Immunohistochemical results. Cases with median (**A**) and highly abundant (**B**) intraepithelial CD8^+^ T cells in colorectal carcinoma. Scale bar, 50 *μ*m.

shows one of the cases of colorectal carcinoma showing the median number of intraepithelial CD8^+^ T cells. The distribution of the number of T cells was quite asymmetric (Figure 2A

**Figure 2:**
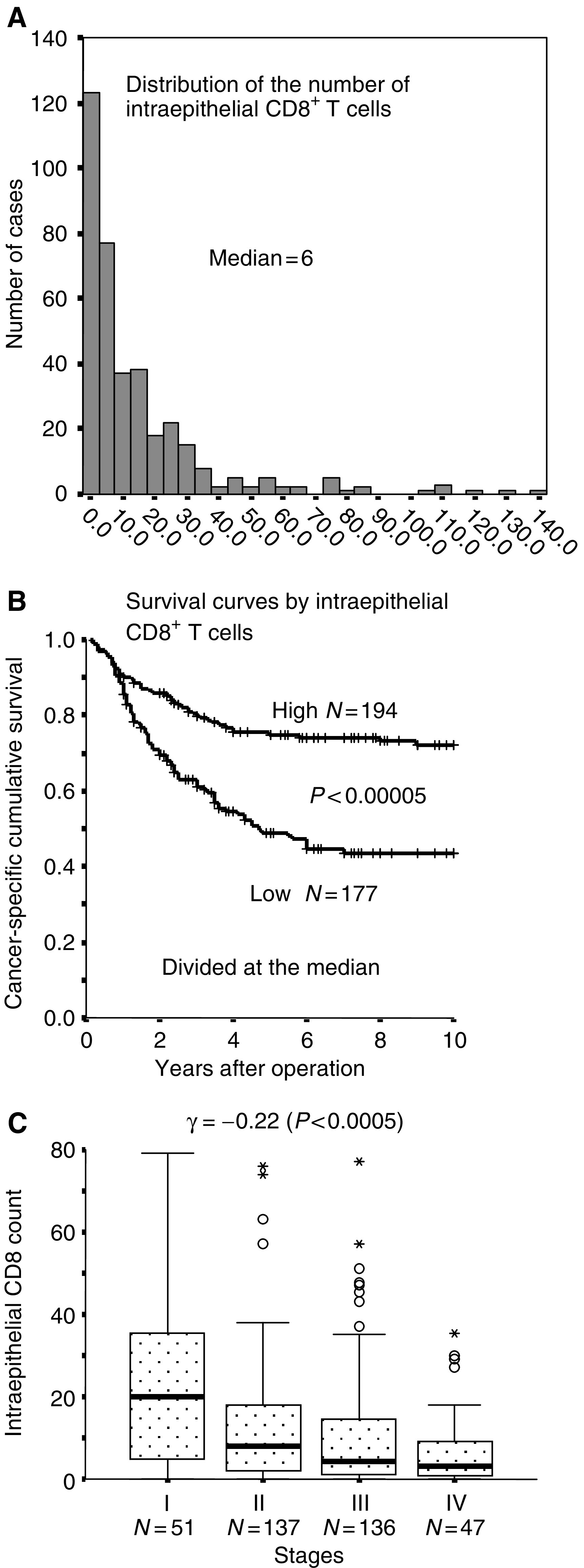
Distribution histogram of the number of intraepithelial CD8^+^ T cells in colorectal carcinoma (**A**), showing a distinct asymmetric pattern. Cancer-specific survival curves by intraepithelial CD8^+^ T cells in all patients (divided into two at the median) (**B**). The longitudinal axis, cancer-specific cumulative survival rate. The horizontal axis, years after operation. Note an inverse correlation between the number of intraepithelial CD8^+^ T cells and the stages (**C**), depicted by Box–Whisker plot showing 25 percentile, median, and 75 percentile.

). Some cases showed abundant intraepithelial CD8^+^ T cells as exemplified in Figure 1B, but such cases were quite infrequent. For the statistical analyses we divided the patients into two groups at the median number. Univariate analysis in all 371 cases revealed that intraepithelial CD8^+^ T cells (Figure 2B) and peritumoral tumour-infiltrating lymphocytes (TILs), together with stage and invasion pattern, were significant for cancer-specific survival (Table 2

**Table 2:** Results of univariate analysis

		***P*-value (log-rank test)**	
**Parameters**	**Risk factor**	**All patients**	**Curative operation group**
**Stage**	More advanced stage	<0.00005	<0.00005
**Invasion pattern**	Infiltrating pattern	<0.00005	<0.00005
***Intraepithelial CD8+ T cells***	*Low*	<0.00005	<0.00005
**Peritumoral TILs**	Not conspicuous	<0.00005	0.0002
Differentiation	(Poor differentiation)	0.066	0.97
MMR proteins	(No abnormality)	0.17	0.083
Site	(Rectum)	0.35	0.1
Age		0.68	0.93
Gender		0.97	0.95

Bold face parameters significant for cancer-specific survival. MMR proteins, mismatch repair proteins (hMLH1 and hMSH2).

). Histologic differentiation, age, gender, site of the primary tumour, or abnormality in mismatch repair proteins were not significant. The number of intraepithelial CD8^+^ T cells inversely correlated with the stage (Figure 2C), and it was mildly higher in the right-sided colon than in the left-sided colon or rectum without statistical significance (*P*=0.43 by Kruskal–Wallis test). The lack of the expression of mismatch repair proteins was detected in 36 of 366 cases. Five cases were excluded due to lack of reactivity for MMR proteins in non-neoplastic cells. This lack was not associated with a significant increase in the number of intraepithelial T cells (*P*=0.21 by Mann–Whitney test). The number of intratumoral CD8^+^ T cells positively correlated with peritumoral TILs (*γ*=0.35, *P*<0.01).

Closer inspection of survival curves by the number of intraepithelial CD8^+^ T cells (divided at the median) revealed a distinctive feature: the two curves were nearly the same during the first 1 year, and became gradually differentiated after 2 years postoperation (Figure 2B). To further analyse this feature, we compared the results of multivariate analyses set at different observation periods. As shown in Table 3

**Table 3:** Results of multivariate analyses by logistic model at 5- and 2-year observation periods

**5-year observation period**							
**All patients (*N*=295)**				**Curative operation group (*N*=233)**			
**Parameters (risk factor)**	**Odds ratio**	**95% CI**	***P***	**Parameters**	**Odds ratio**	**95% CI**	***P***
**Lymph node metastasis**^a^ (positive)	8.3	4.3–16	<0.0005	**Lymph node metastasis**^a^	5.9	3.0–12	<0.0005
**Distant metastasis**^a^ (positive)	230	29–1800	<0.0005	**Invasion pattern**	3.3	1.6–6.5	0.001
**Invasion pattern** (infiltrating)	3.2	1.6–6.0	0.001	***Intraepithelial CD8^+^ T cells***	*2.4*	*1.2*–*4.8*	*0.011*
***Intraepithelial CD8^+^ T cells*** *(low)*	*2.3*	*1.2*–*4.3*	*0.01*				
							
Parameters not significant^b^: peritumoral TILs (*P*=0.25, 0.24), differentiation (*P*=0.47, 0.79), site (*P*=0.86, 0.78), MMR proteins (*P*=0.99, 0.81), age (*P*=0.20, 0.26), gender (*P*=0.77, 0.69), invasion to subserosa^a^ (*P*=0.37, 0.41)							
							
**2-year observation period**							
**All patients (*N*=348)**				**Curative operation group (*N*=290)**			
**Parameters**	**Odds ratio**	**95% CI**	***P***	**Parameters**	**Odds ratio**	**95% CI**	***P***
**Lymph node metastasis**^a^	4.3	1.9–9.9	<0.0005	**Lymph node metastasis**^a^	2.4	0.98–5.6	0.056
**Distant metastasis**^a^	55	19–158	<0.0005	**Invasion pattern**	11	3.5–32	<0.0005
**Invasion pattern**	7.2	3.4–15	<0.0005				
							
Parameters not significant^b^: intraepithelial CD8+ T cells (*P*=0.28, 0.63), differentiation (*P*=0.45, 0.87), peritumoral TILs (*P*=0.10, 0.22), site (*P*=0.64, 0.55), MMR proteins (*P*=0.38, 0.65), age (*P*=0.33, 0.71), gender (*P*=0.67, 0.79)							

Note that intraepithelial CD8+ T cell was not significant at 2-year follow-up period. 95% CI=95% confidence interval of Odds ratio. Death of the primary cancer defined as the event. Bold face=parameters significant for patients' survival after stepdown parameter selection analysis. Italic values stress intraepithelial CD8+ T cells. Data input as follows: Lymph node and distant metastases, negative 1 and positive 2; invasion, expanding 1 and infiltrating 2; CD8, high 1 and low 2; TILs, continuous 1 and not continuous 2; differentiation, not poorly 1 and poorly 2; MMR, normal 1 and lack 2; site, rectum 1, left 2 and right 3; gender, male 1 and female 2; age younger 1 and elder 2. CD8 divided at the median, lower 1 and higher 2. Invasion to subserosa, negative 1 and positive 2.

a Analysed in comparison with stage 1.

b *P*-values in the parentheses: The former for all patients and the latter for curative operation group.

(upper part), the number of intraepithelial CD8^+^ T cells was significant for patients' survival at the 5-year observation periods both in all patients, and in the curative operation group by a logistic model. Other parameters significant included stage-related parameters (lymph node and distant metastases) and invasion pattern. The impact (Odds ratio) of intraepithelial CD8^+^ T cells was smaller than that of stage-related factors, which may be caused by the negative correlation with stage (Figure 2C). We also checked the impact of the number of these T cells at other dichotomy points including 70, 75, and 80 percentile values, with none of them being significant (data not shown). In contrast to this, the number of intraepithelial CD8^+^ T cells was not significant at the 2-year observation period by logistic model (Table 3, lower part).

Analyses by Cox proportional hazards models also revealed the same results: the number of these CD8^+^ T cells was significant for patients' survival only when the observation period was set at 2–10 years after operation, but not at 0–2 years in both all patients'- and curative operation groups (Table 4

**Table 4:** Multivariate analyses by Cox proportional hazard model at 0–2 and 2–10 year observation periods

**Late observation period/2–10 years**							
**All patients (*N*=272)**				**Curative operation group (*N*=260)**			
**Parameters (risk factor)**	**Hazard ratio**	**95% CI**	***P***	**Parameters**	**Hazard ratio**	**95% CI**	***P***
**Lymph node metastasis**^a^ (positive)	5.6	3.4–9.3	<0.0005	**Lymph node metastasis**^a^	4.3	2.6–7.4	<0.0005
**Distant metastasis**^a^ (positive)	18	9.8–32	<0.0005	**Invasion pattern**	2.6	1.6–4.4	<0.0005
**Invasion pattern**(infiltrating)	2.1	1.4–3.2	<0.0005	***Intraepithelial CD8^+^ T cells***	*1.8*	*1.1*–*3.0*	*0.015*
***Intraepithelial CD8^+^ T cells****(low)*	*1.4*	*1.0*–*2.1*	*0.048*				
							
Parameters not significant^b^: differentiation (*P*=0.24, 0.95), peritumoral TILs (*P*=0.20, 0.31), site (*P*=0.55, 0.28), MMR proteins (*P*=0.99, 0.87), age (*P*=0.63, 0.57), gender (*P*=0.72, 0.51), invasion to subserosa^a^ (*P*=0.44, 0.53)							
							
**Early observation period/0–2 years**							
**All patients (*N*=359)**				**Curative operation group (*N*=297)**			
**Parameters**	**Hazard ratio**	**95% CI**	***P***	**Parameters**	**Hazard ratio**	**95% CI**	***P***
**Lymph node metastasis**^a^	3.3	1.5–7.4	0.003	**Invasion pattern**	9.9	3.4–29	<0.0005
**Distant metastasis**^a^	17	7.6–37	<0.0005				
**Invasion pattern**	3.3	1.8–6.0	<0.0005				
**Peritumoral TILs**	3.6	1.2–11	0.022				
**Differentiation**	2.4	1.1–5.2	0.03				
Parameters not significant: intraepithelial CD8+ T cells (*P*=0.81), site (*P*=0.71), MMR proteins (*P*=0.31), age (*P*=0.50), gender (*P*=0.76)				Parameters not significant: lymph node metastasis^a^ (*P*=0.12), intraepithelial CD8+ T cells (*P*=0.79), peritumoral TILs (*P*=0.10), differentiation (*P*=0.90), site (*P*=0.81), MMR proteins (*P*=0.74), age (*P*=0.90), gender (*P*=0.33)			

Note that the number of intraepithelial CD8+ T cells was not significant at a 0–2 year observation period. 95% CI=95% confidence interval of hazard ratio. Bold face=parameters significant for patients' survival after stepdown parameter selection analysis. Italic values stress intraepithelial CD8+ T cells. Data input, the same as in Table 2.

a Analysed in comparison with stage 1. Death of the primary cancer defined as the event.

b *P*-values in the parentheses: the former for all patients and the latter for curative operation group.

). These data clearly indicated that the effects of intraepithelial CD8^+^ T cells became evident only through a longer follow-up period.

**Figure 3:**
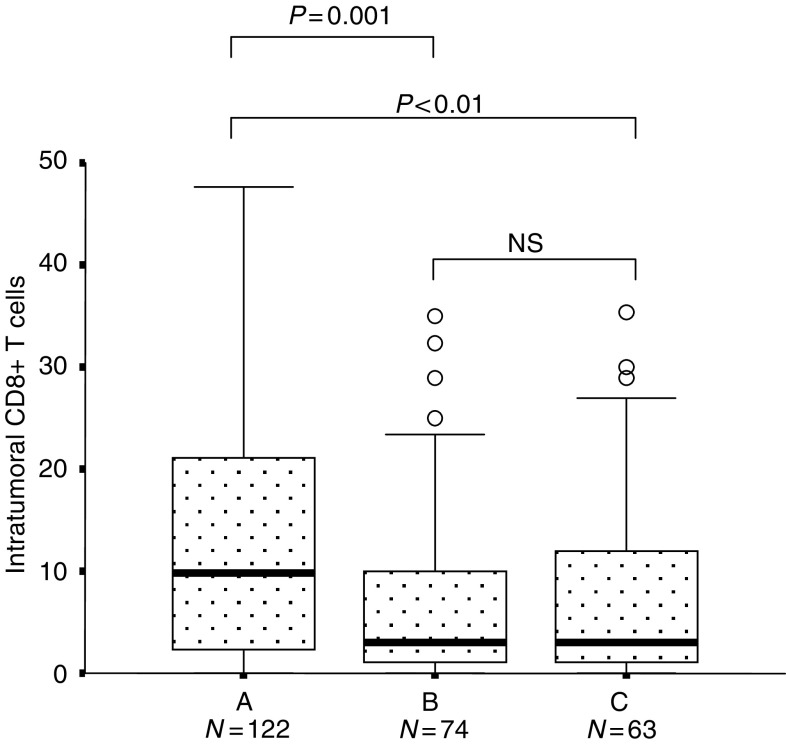
Morphometric results of the number of intraepithelial CD8^+^ T cells among different groups. The longitudinal axis, the number of intraepithelial CD8^+^ T cells. A, Patients alive for more than 5 years (stage I excluded). B, Patients who died of the primary disease after curative operation (stages II and III). C, patients who received noncurative operation (stage IV+stage III with unresectable lymph node metastasis). We excluded cases positive for cancer cells at the resection margins. Overall differences, *P*<0.0005 (Kruskal–Wallis).

To check the above-mentioned results from a different angle, we compared the number of intraepithelial CD8^+^ T cells among patients who were alive for more than 5 years after a curative operation (group A, 122 cases – stage I excluded due to its low mortality), patients who died of the primary disease after a curative operation (group B, 72 cases), and patients who died of the primary disease after non-curative operation (group C, 63 cases). As shown in Figure 3

, the number of intraepithelial CD8^+^ T cells in group A was significantly higher than those in groups B and C, without any significant differences between groups B and C. These data were consistent with the notion that intraepithelial CD8^+^ T cells are a prognostic factor, and further suggested that the decrease of these T cells in stage IV may not be a consequence of systemic immunosuppression by widely spread cancer (for details, see Discussion).

**Figure 4:**
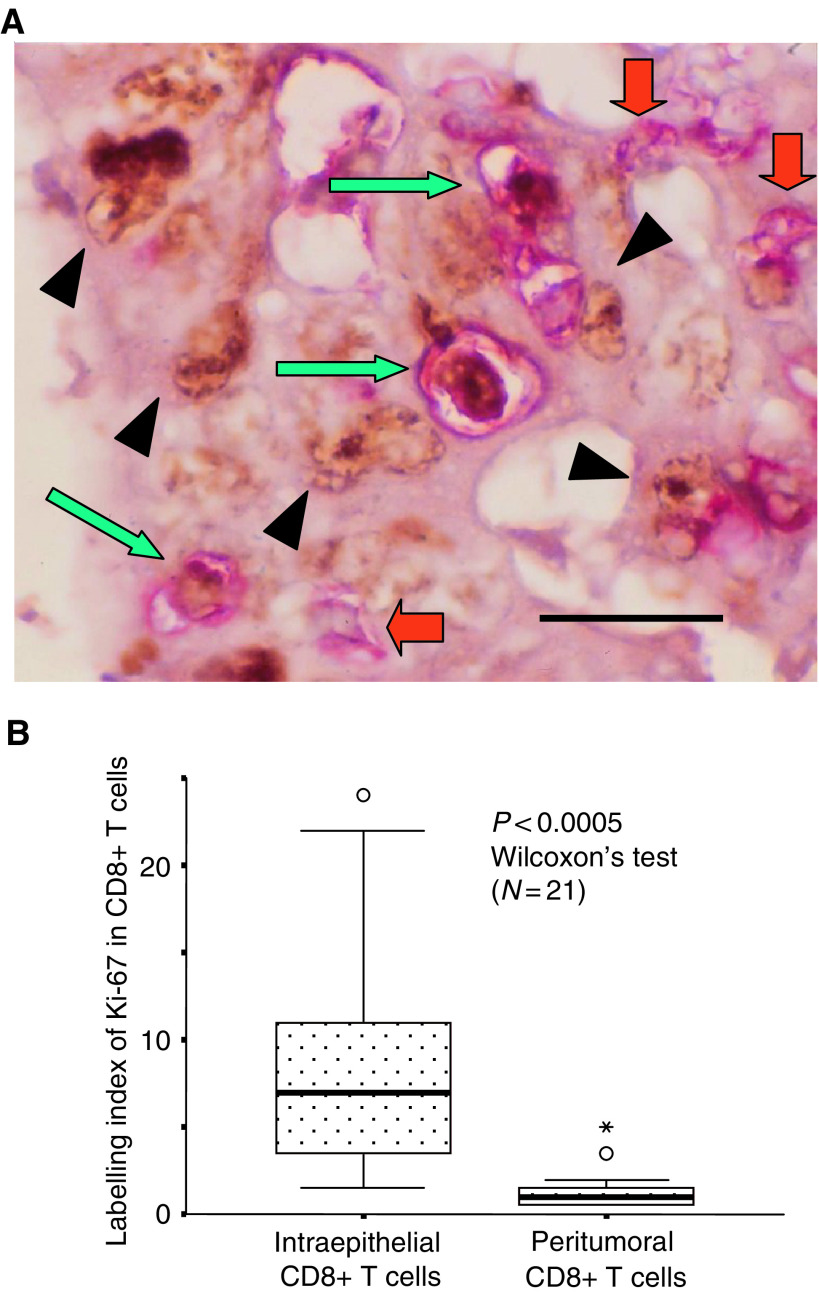
Double staining for CD8 (red) and Ki-67 (brown) (**A**). Green arrows indicate double positive cells (nucleus labelled in brown, cell surface in red). Red arrows indicate CD8+ T cells without labelling for Ki67. Arrowheads indicate single positivity for Ki-67 in cancer cells. Scale bar, 20 *μ*m. Comparison of the labelling index of Ki-67 between intraepithelial and peritumoral CD8^+^ T cells (**B**).

We next estimated the proliferative activity of T cells by their labelling index for Ki-67 by double-labelling immunohistochemistry (Figure 4A

), and compared this between intraepithelial- and peritumoral CD8^+^ T cells in 21 representative cases of well or moderately differentiated adenocarcinoma, of which only one case showed the lack of MMR proteins. Peritumoral T cells refers to T cells located along the invasive margin, which were usually more abundant than intraepithelial T cells (Ohtani *et al*, 1997). As shown in Figure 4B, intraepithelial CD8^+^ T cells showed a much higher labelling index for Ki-67 (7% in median) than peritumoral CD8^+^ T cells did (1% in median), indicating presumable tumour-specific reactivity of intraepithelial T cells.

## DISCUSSION

The present paper confirmed that the number of intraepithelial CD8^+^ T cells, counted at the most densely distributed areas, was an independent prognostic factor in colorectal cancer in a larger-scale study (371 patients), and that this effect was most evident when the patients were divided into two groups at the median number of these T cells. Of particular importance, we newly demonstrated that the effects of these immune cell factors became manifest only after a long follow-up period.

First, it is necessary to summarise why we focused on intraepithelial CD8^+^ T cells. We initially observed Epstein–Barr virus-associated gastric cancer to find an abundance of intraepithelial CD8^+^ T cells (Saiki *et al*, 1996). We then revealed that intraepithelial T cells (=T cells within cancer cell nests) in colorectal cancer had the most significant impact on patients' survival than those in other locations (Naito *et al*, 1998). The present study at an early stage (200 cases) also confirmed that the number of peritumoral CD8^+^ T cells was not significant for patients' survival by multivariate analysis (Naito *et al*, unpublished data). Also, the number of peritumoral CD68^+^ macrophages and that of peritumoral neutrophils were not significant even under univariate analysis (Naito *et al*, unpublished data). Therefore, we focused on intraepithelial CD8^+^ T cells in the present study (371 cases).

The main causes of death after curative operation are metachronous metastases in distant organs, lymph nodes, or peritoneal cavity and recurrence at the primary site. These ominous consequences are generally caused by the growth of micrometastases that were considered to be present at the time of operation. So long as we assume that intraepithelial CD8^+^ T cells attack tumour cells only at the primary site, our results are difficult to explain, because our data showed that the patients' survival was affected by the degree of T-cell responses in the resected primary tumour. Thus, other explanations are required. Here we hypothesise effective suppression of micrometastasis in distant organs or near the primary site in cases with higher numbers of these CD8^+^ T cells. In other words, our data suggest the presence of a systemic immunosurveillance mechanism functioning at sites other than primary tumour, and that this immune activity is in good correlation with the number of CD8^+^ T cells in the primary tumour. Inversely, only insufficient immune mechanisms would have been functioning in cases without such intensive CD8^+^ T-cell responses. These considerations are consistent with the present observation that the number of intraepithelial CD8^+^ T cells is equally low in the cases that died of cancer after a curative operation and in patients who underwent a noncurative operation due to unresectable macrometastases. The latter two situations could be similar from the standpoint of the insufficient host immune responses to control the metastasis.

The higher proliferative activity of intraepithelial CD8^+^ T cells we observed suggests the presence of certain antigenic stimuli to these lymphocytes (Saiki *et al*, 1996). Proliferation of central memory-like CD8^+^ T cells has already been confirmed at the site of inflammation after recall antigenic stimuli (Weninger *et al*, 2001). Therefore, the proliferating T cells we observed would have been primed and differentiated in the secondary lymphoid tissues (i.e. regional lymph nodes), and it is possible to speculate that such presumable effector cells could home into other organs or tissues, along with the primary tumour, and suppress micrometastasis.

We also have to consider the possibility that the decrease in the number of intraepithelial CD8^+^ T cells in cases with synchronous metastasis may be a consequence of systemic immunosuppression (‘consequence theory’). An incoherence emerges with this consequence theory; that is, patients who died of cancer after curative operation, in this scenario, would be expected to have a higher number of intraepithelial CD8^+^ T cells because their metastatic foci are clinically undetectable, being unable to cause systemic immunosuppression at the time of operation (cf. Saito *et al*, 2000). In reality, our data showed no difference in the number of T cells between the curative and noncurative groups. This fact does not support the above ‘consequence theory,’ but suggests that the decrease of intraepithelial T cells might reflect an intrinsic lack of systemic antitumour immune responses.

Microsatellite instability (MSI), as a result of frame shift mutation, is related to a prominent tissue infiltration of T cells in colorectal cancer (Ishikawa *et al*, 2003). In the present study, immunohistochemistry for representative mismatch repair (MMR) enzymes, an alternative method to detect MSI, revealed no significant increase of intraepithelial CD8+ T cells in cases with a loss of MMR eznymes, and no significant increase in right-sided colon cancer, where the incidence of MSI is high. Therefore, factors other than MSI may also be important for abundant T-cell responses in colorectal cancer.

In conclusion, our present study demonstrated the effect of intraepithelial CD8^+^ T cells at longer follow-up periods. This has lead us to the hypothesis that these CD8^+^ T cells may be a indicator of the presence of systemic immunosurveillance that can suppress micrometastasis in other sites as well as tumour suppression in the primary sites. Considering possible clinical applications, further studies are required to analyse these presumptive immune effector mechanisms.

## References

[bib1] Albert ML, Darnell RB (2004) Paraneoplastic neurological degenerations: keys to tumour immunity. Nat Rev Cancer 4: 36–44 14708025 10.1038/nrc1255

[bib2] Dolcetti R, Viel A, Doglioni C, Russo A, Guidoboni M, Capozzi E, Vecchiato N, Macri E, Fornasarig M, Boiocchi M (1999) High prevalence of activated intraepithelial cytotoxic T lymphocytes and increased neoplastic cell apoptosis in colorectal carcinomas with microsatellite instability. Am J Pathol 154: 1805–1813 10362805 10.1016/S0002-9440(10)65436-3 PMC1866613

[bib3] Dunn GP, Bruce AT, Ikeda H, Old LJ, Schreiber RD (2002) Cancer immunoediting: from immunosurveillance to tumor escape. Nat Immunol 3: 991–998 12407406 10.1038/ni1102-991

[bib4] Fukuzawa K, Takahashi K, Furuta K, Tagaya T, Ishikawa T, Wada K, Omoto Y, Koji T, Kakumu S (2001) Expression of fas/fas ligand (fasL) and its involvement in infiltrating lymphocytes in hepatocellular carcinoma (HCC). J Gastroenterol 36: 681–688 11686478 10.1007/s005350170031

[bib5] Hamilton SR, Aaltonen LA (eds) (2000) WHO classification of tumours. Pathology and genetics of tumours of the digestive system. Lyon: IARC press

[bib6] Henderson RA, Finn OJ (1996) Human tumor antigens are ready to fly. Adv Immunol 62: 217–256 8781270 10.1016/s0065-2776(08)60431-9

[bib7] Ishikawa T, Fujita T, Suzuki Y, Okabe S, Yuasa Y, Iwai T, Kawakami Y (2003) Tumor-specific immunological recognition of frameshift-mutated peptides in colon cancer with microsatellite instability. Cancer Res 63: 5564–5572 14500396

[bib8] Jass J, Cottier DS, Jeevaratnam P, Pokos V, Holdaway KM, Bowden ML, Van de Water NS, Browett P (1995) Diagnostic use of microsatellite instability in hereditary non-polyposis colorectal cancer. Lancet 346: 1200–1201 7475662 10.1016/s0140-6736(95)92902-9

[bib9] Jass JR, Love SB, Northover JM (1987) A new prognostic classification of rectal cancer. Lancet 1(8545): 1303–1306 2884421 10.1016/s0140-6736(87)90552-6

[bib10] Khong HT, Restifo NP (2002) Natural selection of tumor variants in the generation of ‘tumor escape’ phenotype. Nat Immunol 3: 999–1005 12407407 10.1038/ni1102-999 PMC1508168

[bib11] Melief CJ, Toes RE, Medema JP, van der Burg SH, Ossendorp F, Offringa R (2000) Strategies for immunotherapy of cancer. Adv Immunol 75: 235–282 10879286 10.1016/s0065-2776(00)75006-1

[bib12] Menon AG, van Rhijn CMJ, Morreau H, Putter H, Tollenaar RAEM, van de Velde CJH, Fleuren GJ, Kuppen PJK (2004) Immune system and prognosis in colorectal cancer: a detailed immunohistochemical analysis. Lab Invest 84: 493–501 14968119 10.1038/labinvest.3700055

[bib13] Michael-Robinson JM, Biemer-Huttmann A, Purdie DM, Walsh MD, Simms LA, Biden KG, Young JP, Leggett BA, Jass JR, Radford-Smith GL (2001) Tumour infiltrating lymphocytes and apoptosis are independent features in colorectal cancer stratified according to microsatellite instability status. Gut 48: 360–366 11171826 10.1136/gut.48.3.360 PMC1760146

[bib14] Naito Y, Saito K, Shiiba K, Ohuchi A, Saigenji K, Nagura H, Ohtani H (1998) CD8+ T cells infiltrated within cancer cell nests as a prognostic factor in human colorectal cancer. Cancer Res 58: 3491–3494 9721846

[bib15] Nakano O, Sato M, Naito Y, Suzuki K, Orikasa S, Aizawa M, Suzuki Y, Shintaku I, Nagura H, Ohtani H (2001) Proliferative activity of intratumoral CD8(+) T-lymphocytes as a prognostic factor in human renal cell carcinoma: clinicopathologic demonstration of antitumor immunity. Cancer Res 61: 5132–5136 11431351

[bib16] Ohtani H, Naito Y, Saito K, Nagura H (1997) Expression of costimulatory molecules B7-1 and B7-2 by macrophages along invasive margin of colon cancer: a possible anti-tumor immunity? Lab Invest 77: 231–241 9314947

[bib17] Rosenberg SA (2001) Progress in human tumour immunology and immunotherapy. Nature 411: 380–384 11357146 10.1038/35077246

[bib18] Saiki Y, Ohtani H, Naito Y, Miyazawa M, Nagura H (1996) Immunophenotypical characterization of Epstein–Barr virus-associated gastric carcinoma: massive infiltration by proliferating CD8^+^ T lymphocytes. Lab Invest 75: 67–76 8683941

[bib19] Saito K, Takeha S, Shiiba K, Matsuno S, Sorsa T, Nagura H, Ohtani H (2000) Clinicopathologic significance of urokinase receptor- and MMP-9-positive stromal cells in human colorectal cancer: functional multiplicity of matrix degradation on hematogenous metastasis. Int J Cancer 86: 24–29 10728590 10.1002/(sici)1097-0215(20000401)86:1<24::aid-ijc4>3.0.co;2-a

[bib20] Schumacher K, Haensch W, Roefzaad C, Schlag PM (2001) Prognostic significance of activated CD8(+) T cell infiltrations within esophageal carcinomas. Cancer Res 61: 3932–3936 11358808

[bib21] Smyth MJ, Godfrey DI, Trapani JA (2001) A fresh look at tumor immunosurveillance and immunotherapy. Nat Immunol 2: 293–299 11276199 10.1038/86297

[bib22] Sobin LH, Wittekind C (eds) (2002) TNM classification of malignant tumours 6th edn. New York: Wiley-Liss

[bib23] Weninger W, Crowley MA, Manjunath N, von Andrian UH (2001) Migratory properties of naive, effector, and memory CD8T cells. J Exp Med 194: 953–966 11581317 10.1084/jem.194.7.953 PMC2193483

[bib24] Zhang L, Conejo-Garcia JR, Katsaros D, Gimotty PA, Massobrio M, Regnani G, Makrigiannakis A, Gray H, Schlienger K, Liebman MN, Rubin SC, Coukos G (2003) Intratumoral T cells, recurrence, and survivalin epithelial ovarian cancer. N Eng J Med 348: 203–213 10.1056/NEJMoa020177 12529460

